# Risk of a disaster: Risk knowledge, interpretation and resilience

**DOI:** 10.4102/jamba.v12i1.845

**Published:** 2020-05-27

**Authors:** Osamuede Odiase, Suzanne Wilkinson, Andreas Neef

**Affiliations:** 1 Department of Civil and Environmental Engineering, Centre for Disaster Recovery, Resilience, and Reconstruction, The University of Auckland, Auckland, New Zealand; 2 School of Built Environment, Massey University, Auckland, New Zealand; 3 Department of Development Studies, The University of Auckland, Auckland, New Zealand

**Keywords:** risk knowledge, risk perception, natural hazards, disaster preparedness, resilience, South Africa

## Abstract

Knowledge and interpretation of local risks are essential in disaster mitigation. Auckland’s exposure to multiple hazards is a source of national concern. Considering the multiplicity of natural hazards in Auckland, investigations on how communities can enhance their resilience to possible disasters have become imperative. Convincing individuals to embark on activities that would reduce their vulnerability to natural hazards is difficult, especially in communities that have not recently experienced the impact of natural hazards. This research investigated risk knowledge and interpretation in the South African community in Auckland. Data for this study were collected from both primary and secondary sources. A questionnaire was distributed amongst the South African population, and follow-up interviews with participants constituted the primary sources of data collection. Other sources were materials in the public domain. Regarding data analysis, an independent-sample *t*-test and Spearman’s correlation analysis were used to analyse the quantitative research data. A general inductive approach for qualitative data was used to analyse the research interviews. The research confirmed the subjectivity in risk perception and also revealed a high-risk perception, especially for earthquake, flood and tsunami. Whilst this study agreed that there is a relationship between risk perception and preparedness, such relationship is often contextual. The research concludes that risk perception could contribute to disaster resilience if communities appreciate the impact of a natural hazard irrespective of disaster experience or otherwise.

## Introduction

Many type of literature exists on natural hazards in Auckland; the majority of literature is focused on volcanic eruptions. The Auckland volcanic projects (Paton et al. 1999) projecting real-time eruption (Lindsay et al. 2009) and the consequences of eruptions (Magill & Blong 2006) are cases in point. In spite of potential disaster from volcanic eruptions and dangers from other local hazards, evidence from the existing literature on risk perception in Auckland and New Zealand, in general, shows that no investigation has been conducted on how the African community in Auckland understands and reacts to risks. This investigation becomes necessary because of the hazard landscape of Auckland and the importance of risk perception in designing an effective mitigation strategy and improving resilience.

The overarching objective of this research was to investigate awareness and interpretation of risk amongst South Africans in Auckland. The objective was achieved by answering the following questions:

- Which hazards are likely to affect the community?
- Which hazards are likely to affect individuals in the community?
- How does trust affect risk perception in the community?
- How is risk perception in the community personalised?

Subsequent development in this article proceeds with an examination of previous and related works, and this is followed by this study’s data collection and analysis technique, and then this study’s findings and discussion. Lastly, the summary section concludes this study.

### Context of the research

‘Disaster risk’ is often defined as the proclivity to be impacted by natural hazards. The possibility of being impacted by a disaster arises from the interaction between existing vulnerability and local hazards (Blaikie et al. 2014). Whilst inappropriate social and development policies contribute to vulnerability and risk (Oliver-Smith et al. 2016), the exposure and sensitivity of at-risk elements are equally important in disaster causation (Adger 2006). Whilst disaster risk reduction (DRR) and resilience could be enhanced through social equity in development planning (Collins 2018; United Nations Development Programme, Bureau for Crisis Prevention & Recovery 2004), the importance of community risk awareness and acknowledgement of its predisposition to local hazards cannot be underestimated in DRR. The essence of awareness is for a community to have a different orientation towards hazards and it begins to reflect on resilience.

### Risk knowledge and community resilience

Risk knowledge refers to community awareness of disaster risk. It encompasses but not limited to the awareness of the magnitude of risks, local hazards, and exposure, susceptibility and the capacity of elements at risk of local hazards (World Meteorological Organisation 2020). Often than not, risk knowledge provides the needed impetus for community resilience and a psychological boost to community participation in DRR and mitigation (Adger 2006; Allen 2006; McEntire & Myers 2004). Essential aspects of risk knowledge and community resilience are hazard analysis and vulnerability assessment (Bogardi & Birkmann 2004) because they enable the community to design adaptive response to a potential disaster (Cutter et al. 2008). Aside from adaptive capacity, risk knowledge improves adaptive response to local risk through community participation in hazard mapping (Gaillard & Pangilinan 2010). The importance of local participation in hazard mapping is that the community acquires the first-hand knowledge of the spatial information on local hazards and their susceptibility to the risks. Local participation in hazard knowledge also provides the opportunity to collaborate local and scientific knowledge to address community vulnerability to local hazards (Mercer et al. 2009).

In a disaster-prone community, risk knowledge is better informed by knowledge of the potential consequences of disaster rather than the historical occurrence of a disaster (Tierney et al. 2001). Risk knowledge is particularly critical in an urban community where populations migrate from a familiar terrain of known risk to the unfamiliar landscape (Mitchell 1999). The extent to which risk knowledge and participation in hazard management influences personal adjustment to local hazards is closely related to how individuals perceive or interpret their vulnerability to local hazards (Oliver-Smith & Hoffman 1999; Wachinger et al. 2013). Although perception plays a predominant role in personal response to a potential hazard event, it is underpinned by socio-demographic factors.

### Risk interpretation

Whilst it is difficult to quantify risk perception, it could be gauged by the actions people embarked upon in anticipation of a potential disaster from local hazards. Cutter (1993), cited in Murphy et al. (2005:21), explains ‘human perception of risk of a disaster as a process that links individual judgements of the degree of danger (risk) with action (preparedness)’. The assumption that local hazards may impact a community differentiates risk knowledge from risk perception. Underlining the assumption of being affected is the nature and features of local risks, intensity and frequency of individual experience (Kates 1971). These variables were further explained by Fischhoff et al. (1978) and in Slovic and Weber’s (2002) psychometric paradigm. The paradigm recognises dread and newness of a hazard as significant determinants in understanding risk perception from a non-scientific view. In addition to the psychometric paradigm, cultural theorists explore risk perception from a political economy and emotional perspectives (Douglas & Wildavsky 1983). The central theme of their theory is that socio-cultural factors determine how people conceptualise risk. In synergising the above views, Pidgeon et al. (1992:89) posit that risk perception involves personal heuristic on local hazards ‘as well as wider social and cultural values and dispositions that people adopt, towards hazards and their benefits’. Pidgeon and others (1992) accommodate hazard characteristics and the multi-dimensional concept of risk perception.

Under certain circumstances, trusted sources of information and scientific opinion influence judgement on the riskiness of a probable disaster (Eiser et al. 2012; Han, Hörhager & Yan 2017; Paton 2008). Although decision on the riskiness of a hazard may be underpinned by a trusted source of information and scientific advice on risk, the role of protective measures in risk perception cannot be underestimated (Terpstra 2010). People rely on expert knowledge for a rational decision on risk because of the complexity or novelty of particular hazard (Frewer & Salter 2007; Paton 2008). However, the credibility of information, institutional performance in hazard management, and individual experience and satisfaction with previous information determine the level of trust and reliability of risk information (Paton 2008; Paton, Burgelt & Prior 2008a). Aside from trust in human expertise, trust in hard engineering influences risk decisions (Botzen, Aerts & Van den Bergh 2009; Wachinger et al. 2013) as people undermine risk and trust-existing infrastructures to mitigate risks. Although disaster experience and trust play a critical role in risk perception, risk decision is being modified continually by the media and through social interactions amongst individuals (Morgan et al. 2001). This process amplifies or attenuates the perceived risk during personal decision on the risk.

Risk perception is not homogenous amongst individuals. The type of hazard, socio-economic status and demographic factors influence personal judgement on the riskiness of disaster. From the socio-economic perspective, studies have suggested that people prefer to accept risk for economic benefit, rather than suffering from abject poverty in a safer environment (Blaikie et al. 2014; Gaillard, Liamzon & Villanueva 2007). Ruin, Gaillard and Lutoff (2007) argued that the risk of a disaster is often determined not by threats from hazards but by the socio-economic and political constraints that are beyond individual’s control. These limitations are also pivotal in their decisions to personalise the risk, embark on mitigation and preparedness actions or disregard the threat (Wachinger et al. 2013).

Individual choice of action towards risk explains the inconsistencies between the risk perception and response at different spatial and temporal scales. The expectation is for people to respond positively to high-risk judgement. However, this is often not the case, as a personal estimation of risk is subjective and socially constructed (Johnson et al. 2013) as opposed to objectivity. In a socio-ecological environment, the risk is not confined to a simple mathematical model of risk and probability because it undermines the influence of human input in a social phenomenon (Pidgeon et al. 1992). In tandem with this view, Slovic (1999), cited in Botterill and Mazur (2004) and Hewitt (1997), argued that:

[*W*]hat constitutes a risk and the level of perception is socially constructed because risk could not be separated from choices which condition individual beliefs and circumstances; and the complexity of the society. (p. 22)

Whilst some individuals may be willing to respond positively to perceived risk, personal circumstances and feeling towards risk may dictate otherwise. Economic benefit, individual self-delusion about risk and lack of confidence in preparedness measures are some of the factors that undermine risk perception (Eiser et al. 2012; Johnson et al. 2013; Terpstra 2010).

Consequently, predicting disaster preparedness on risk perception poses many challenges, especially in an urban community. The multiple ethnic and cultural constellations and unequal access to social and economic opportunities are some of the challenges to human expectations concerning risk and action. Consequently, the relationship between risk perception and disaster preparedness is not linear (Eiser et al. 2012) because of intervening variables. Notwithstanding the probability of a disaster or severity of impact, people may choose not to be prepared for a variety of reasons. Johnson et al. (2013) and Paton and Johnson (2001) identify lack of confidence and motivation in personal preparation. In some cases, a false sense of safety prevents people from embarking on preparedness activities (Perry, Lindell & Tierney 2001). Thus, understanding urban community’s risk perception and its underpinning factors are crucial for improving risk communications and designing adequate and appropriate response and policies towards risk and hazard management (Grothmann & Reusswig 2006; Xu et al. 2014).

## Study area and methodology

### Area of the study

Auckland in the North Island of New Zealand is located on latitude 36.848461 S and longitude 174.763336 E (Longlat.com 2020:1). It is the fastest-growing multi-cultural city in the country. More than 90% of Auckland’s population lives in urban areas. Unlike the South Island city of Christchurch and its environs, Auckland is least susceptible to earthquake because of its location on the Australian tectonic plate, 300 km–500 km northwest of the active plate boundary of the Australian and Pacific plates (Auckland Council 2015:6). However, it is most prone to volcanic eruptions and coastal erosion because of its location on the Auckland Volcanic Field (AVF) covering 100 km^2^ of the urban areas and approximately 3000 km length of the coastal shoreline (Auckland Council 2015:6). Apart from earthquakes and volcanic eruptions, Auckland is also highly vulnerable to a wide range of multiple hazard impacts from severe weather events, floods, tsunamis and landslides.

### The community of this study

The office of Statistics New Zealand (2015) recorded the population of South Africans in Auckland to be 30 612 as of the 2013 census. Whilst the history of South African migration to New Zealand dates to the 19th century, a large-scale emigration wave from South Africa started after the demise of the Apartheid regime in the early 1990s (Walrond 2015). By 2013, the South African community grew to be the fifth largest community in Auckland and New Zealand (Statistics New Zealand 2013). Although the community is divided ethnically, it identifies itself as African.

Notwithstanding that most of the South African population lives in the North shore of Auckland, this research considers the community as a dispersed community as other members of the community live outside the north of Auckland. The South African community, like other communities in Auckland, faces the risk of multiple disasters. Whilst most of the community members may be highly prone to coastal hazards because of residential location, they are equally prone to volcanic eruptions because of the location of Auckland.

**FIGURE 1:**
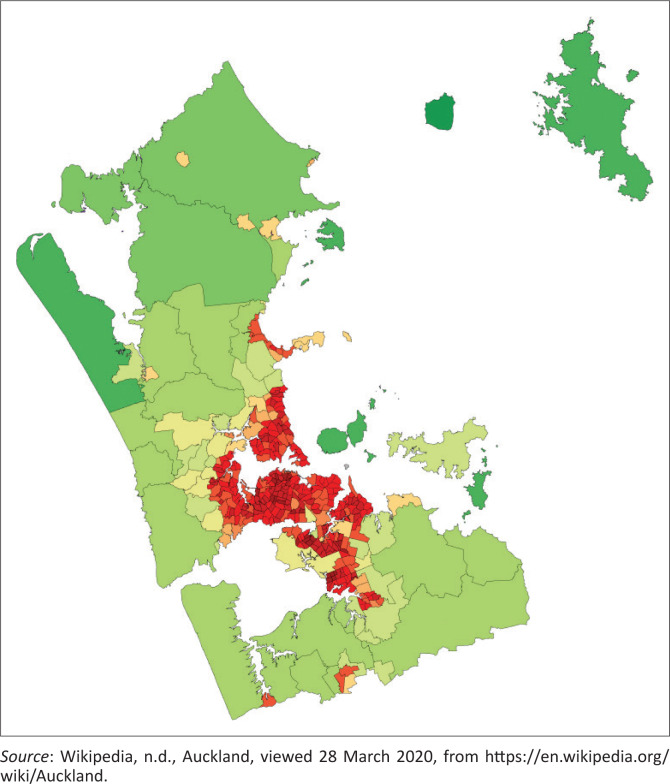
Map of Auckland.

This research constitutes a part of the ‘Resilient Urban Communities Project’ (2015–2019), Resilience to Nature’s Challenges funded by the New Zealand Government through the National Science Challenge. One of the stakeholders in this project is the Auckland City Council. In collaboration with the Auckland Council, a criterion was set out for identifying proxy communities for the African community: firstly, a community with longevity and roots in Auckland, and secondly, a diverse community. Regarding diversity and longevity in Auckland, the South African community fitted into that matrix.

## Data method: Collection and analytical process

The primary objective of this study was to investigate how the South African community in Auckland interprets the risk of disasters from natural hazards. In order to achieve this objective, this study used a mixed-method to collect data from members of the community. Questionnaire and interviews were the primary sources of data. These sources were complemented by existing literature as secondary data. The questionnaire protocol consisted of sections relating to the socio-demographic background of participants, risk perceptions, trust and risk and disaster readiness. A sizeable proportion of the 150 questionnaires were distributed to adult South Africans aged 18 years and above between October 2016 and December 2017 in a church in the North Shore neighbourhood of Auckland. One of the church leaders assisted in the distribution and collection of the questionnaire. The questionnaire was also distributed with the help of two research assistants in hospitals, government ministries and other areas with a sizeable population of South Africans in Auckland. Of the 150 questionnaires that were distributed, 88 questionnaires were completed and returned by participants. The returned rate was about 58%, a little more than the minimum requirement of 82 participants for correlation analysis and two-tailed hypotheses (Onwuegbuzie & Collins 2007). The returned questionnaire for this study indicated that 48% males and 52% females completed the questionnaire. In all, 89% of this study’s participants were between the working age of 18 and 65 years, whilst only 11% constituted a dependent population. Whereas 62% of the respondents had New Zealand citizenship, only 2% had lived in Auckland for more than 26 years; 96% of the respondents had secondary and higher education.

In addition to the questionnaire, this study conducted five face-to-face and three telephonic interviews with community members that indicated their interest for an interview in the questionnaire. The purpose of the interviews was to have further discussion on the findings of the questionnaire. This study used both independent-sample *t*-test and Spearman’s correlation coefficient to analyse questionnaires to examine gender difference in risk perception and the relationship between independent and dependent variables used in this research. Thomas’s (2006) General Inductive Approach to qualitative analysis for qualitative data in conjunction with a three-step coding cycle as explained by Saldaña (2013) was used to select initial data codes, categories and overarching themes. This study discussed findings in line with the existing theoretical propositions on risk perception.

### Ethical considerations

Ethical clearance to conduct this study was obtained from the University of Auckland Human Participants Ethics Committee (Reference No. 017500).

## Findings and discussion

### Disaster risk perception and awareness

The participants were asked whether they knew the hazards landscape of Auckland and hazards that could affect Auckland. The question was asked because knowledge of hazards determines the coping and adaptive capacities to be adopted. Responses from the participants indicated high awareness and the likelihood of disasters from one or more hazards in Auckland. The likelihood of floods, earthquake and tsunami was considered high by the respondents, whilst hazards such as volcanic eruptions, landslide and tornado were deemed least likely to occur (Table 1).

**TABLE 1:** Community risk awareness and perception: Descriptive summary results for hazards likely to affect Auckland.

Natural hazard	Not likely, % (*N* = 88)	Likely, % (*N* = 88)
Earthquake	33	67
Flooding	22	78
Tsunami	33	67
Volcanic eruptions	44	56
Landslide	58	42
Coastal erosion	35	65
Tornado	58	42

Regarding the hazards under consideration, the community’s sense of danger was more towards earthquake, flooding and tsunami than other hazards in the community. Disaster from a tornado was considered least likely to happen in the community. Participants’ responses were underpinned by hazard information and location. The high level of risk attached to floods by the community was not related to previous experience but to the geographical location of most of the participants’ residences in the north shore of Auckland. Earlier research conducted by Heitz et al. (2009) and Kaiser and Witzki (2004) has supported the association between the location of residence and risk perception. Whilst Auckland may not be susceptible to earthquakes because of its location on the Australia tectonic plate, participants’ perception of the risk of earthquakes in Auckland was likely to be influenced by events in other parts of the country, specifically Christchurch and Wellington and the information available on the hazards. Sixty-seven per cent of the research participants felt that a tsunami would affect Auckland. A similar percentage was also recorded for an earthquake because of its trigger effect on tsunami and associated information that are available to participants from the local emergency management agency and through media amplification of the risk of a tsunami (Kasperson et al. 1988). The perception of the risk of volcanic eruptions in Auckland was low in comparison to earthquakes and tsunami, which were perceived by 67% participants. Interviews with research participants revealed that they dread earthquakes and tsunami because these were much talked about in Auckland than volcanic eruptions, and, moreover, because of the less occurrence of a disaster arisen from the hazard and the time lag between one occurrence and another (Kajihara & Kishimoto 2011). The level of risk perception for earthquake indicated that little or no social learning has transpired between the migrant community and the local agency. Because only 2% of the community has lived in Auckland for 26 years and more, most members of the community were unaware of the risk and past activities relating to earthquakes. The research results have implications on emergency planning and resilience in Auckland. The community is less likely to be prepared for a possible volcanic eruption in Auckland even though Auckland is more susceptible to volcanic eruptions than other hazards. Similarly, other hazards in Auckland were likely to be undermined in resilience planning by the community.

### Individual perception of risk

To further understand risk perception amongst the South African community, participants were asked to respond on a 5-point Likert scale on the likelihood of being impacted by local hazards (Table 2). The essence of the question was to know whether individual risk perception differed in the community. This study’s findings suggest a slight contrast between how the community and individual households perceived risks. Whilst disasters from flood, earthquakes and tsunami were worrisome for the community, the danger of tsunami was not considered by most individuals (*M* = 2.42, *SD* = 1.21) to impact them. Instead, participants considered volcanic eruptions as the second major disaster that could impact individuals after an earthquake. This consideration for volcanic eruptions constituted a major difference between how the community perceived volcanic eruptions and household perception of the hazard. Individual risk perception for volcanic eruptions surprisingly was high (*M* = 2.77, *SD* = 1.36) because 96% of the study population had no previous experience of volcanic eruptions’ hazard. Although 56% of the community believed that volcanic eruptions could happen in Auckland, the health implications of volcanic eruptions and its impact on daily activities were the reasons for high-risk perception amongst individual households.

**TABLE 2:** Mean summary: Perception of risk and the likelihood of individuals being affected by natural hazards.

Natural hazard	Mean	Standard deviation
Earthquake	2.86	1.35
Flooding	2.65	1.22
Tsunami	2.42	1.21
Volcanic eruptions	2.77	1.36
Landslide	2.25	1.27
Coastal erosion	2.08	1.26
Tornado	2.23	1.21

Likelihood of being impacted by local hazards on 5-point Likert scale: 1 = not likely, 2 = somewhat likely, 3 = moderate likely, 4 = quite likely, 5 = high likely.

The reason for high-risk perception for earthquakes amongst households was the perceived likelihood of occurrence. Individuals in the community were worried because they felt that earthquakes were a national problem that could affect them, irrespective of location. The unpredictability of an earthquake in comparison to other hazards was also a source of concern for the people. However, participants’ apprehension for the earthquake was misplaced because the geographical location of Auckland made it less susceptible to an earthquake. The apparent reason for the apprehension could be related to the occurrence of earthquake events in other areas of the country and the publicity attached to damage caused by earthquakes. The expected risk from flooding was equally high, but to a lesser degree when compared to other geophysical hazards. Although Auckland is prone to weather events and coastal erosion, their less severe impacts and low incidence could explain the low-risk perception as expressed by individuals. Household perception of risk had implications for disaster emergency plan and participation in pre-disaster activities. Households were likely to embark on disaster plan and participate in a drill that could be unrelated to local hazards.

To further understand individual risk perception in the community, an independent-sample *t*-test and Spearman’s correlation analyses were conducted to examine socio-demographic differences and relationships in risk perception. The mean summary report did not indicate a significant difference between genders. Although a marginal difference existed in their assessment of risks across hazards in Auckland, gender was not influential in risk interpretation in the community. This finding was in line with the previous study by Roder et al. (2016) regarding perception and awareness of landslide and flood risks in Taiwan. Spearman’s correlation analysis conducted to investigate the relationship and direction between age, income, level of education and income across hazards in Auckland showed that education was significantly related to how individuals perceived the risk of tsunami in Auckland (*r*= −0.259 *p* < 0.005) (Table 3). The negative correlation denotes an inverse relationship between education and the perception of risk of tsunami. This finding suggested that a higher educated population had a lower risk perception. This result was, however, not surprising as more than 96% of the community had secondary education and above. Individuals in the community did not interpret risk differently, in spite of the difference in the number of years they had resided in Auckland. The result confirmed the previous studies by Roder et al. (2016) regarding natural hazards and risk perception in Taiwan. Besides education, there was no significant relationship between income, age and years of residence and hazards’ risk perception. Although income varies in Auckland, it did not correlate with risk perception. The previous work conducted by Qasim et al. (2015) has reached a similar conclusion regarding the flood-prone province of Pakistan. In addition to income variable, the research findings supported Qasim and others (2015) on the absence of a relationship between age and risk perception.

**TABLE 3:** Summary of correlations between social variables and perception of hazard risks.

Dependent variables: Natural hazard	Independent variables			
	Number of years in Auckland	Education	Age	Income
Earthquake	0.020	−0.019	0.107	−0.182
Flooding	−0.147	−0.061	−0.021	−0.202
Tsunami	0.072	−0.259*	0.059	−0.185
Volcanic eruptions	0.171	−0.084	0.164	−0.099
Landslide	−0.036	−0.048	0.043	−0.211
Coastal erosion	−0.036	0.001	0.018	0.005
Tornado	−0.122	−0.051	0.046	−0.015

Not significant, *p* > 0.05; significant, *p* < 0.05.

* , Correlation is significant at 0.05 level (two-tailed).

### Trust and risk decisions

Regarding factors that could influence personal assessment on the risks of a hazard and subsequent actions, this study asked respondents questions on a 5-point Likert scale regarding trusted information from the local emergency management agency and its influence on individuals’ risk interpretation. The aggregate mean value (*M* = 3.13, *SD* =1.31) indicated that official information on hazards played a pivotal role in the individual interpretation and subsequent action regarding risk in the South African community. Consequently, most people in the community relied on official information in deciding the riskiness of a hazard, as indicated in the high mean value (*M* =3.67, *SD* = 1.21). The high mean value was not surprising as most participants were not sure of what to do during a disaster event. Emergent themes from follow-up interviews tended to support this study’s quantitative data. It emerged that participants’ limited knowledge of risk interpretation was a primary reason that they relied on trusted information. The limitation arose out of lack of disaster experience, as about 96% of the study population did not have any disaster experience. The conclusion mirrored previous literature and findings by Njome et al. (2010), Visschers and Siegrist (2008) and Wachinger et al. (2013), in which they argued that communities without previous disaster experience and complexity of natural hazards had increased trust in official and expert information. The influence of official information on risk perception varied across hazards. The variation was closely aligned with how participants perceived the risks that could likely affect them.

Table 4 suggests that how information on earthquake, flood and tsunami is likely to influence individuals’ risk perception than other hazards in the locale.

**TABLE 4:** Descriptive summary results of the influence of trusted information on hazard risk perception.

Natural hazard	Mean	Standard deviation
Earthquake	3.73	1.23
Flooding	3.44	1.25
Tsunami	3.78	1.25
Volcanic eruptions	3.36	1.20
Landslide	2.55	1.32
Coastal erosion	2.40	1.25
Tornado	2.47	1.27

Likelihood of being impacted by trusted information on hazard risk perception on 5-point Likert scale: 1 = not likely, 2 = somewhat likely, 3 = moderate likely, 4 = quite likely, 5 = high likely.

Although evidence in Table 4 shows how people’s perception of risk of hazards are likely to be influenced by official information, this study conducted Spearman’s rank-order correlation to determine the direction and relationship between risk information and the perception of risk. The output indicated a positive relationship between risk information and earthquake, flood, tsunami, coastal erosion, volcano and tornado (Table 5). The influence of risk information on hazards was more substantial regarding earthquake, flood and tsunami than other hazards. The results indicated that individual perceptions of risks were positively related to risks that could likely impact them. The levels of correlation further confirmed participants’ fear of hazards and risk information.

**TABLE 5:** Summary of correlations between trusted information and influence risk perception.

Natural hazard	Trusted information
Earthquake	0.289**
Flooding	0.431**
Tsunami	0.383**
Volcanic eruptions	0.239*
Landslide	0.207
Coastal erosion	0.267*
Tornado	0.276**

Not significant, *p* > 0.05; significant, *p* < 0.05.

* , Correlation is significant at 0.05 level (two-tailed).

** , Correlation is significant at 0.01 level (two-tailed).

The work conducted by Arlikatti, Lindell and Prater (2007) and Bronfman et al. (2015) supported the above correlations. Whilst the former found trust in official information to be influential in seismic behavioural and hazard adjustment in the USA, the later found that trusted information from authorities was a strong predictor of how Chileans perceived environmental hazards. However, studies conducted by Dow and Cutter (2000) regarding Hurricane Floyd in South Florida and Paton et al. (2008b) on the effectiveness of information in volcanic perception and adjustment in New Zealand did not align with this study’s findings. The reason could be contextual as risk perception is influenced by disaster experience, information and the personality of people, which differ across at-risk communities.

### Risk perception and personalisation

The degree of danger attached to hazards and the willingness to act on the risk varied from one hazard to another. On a 5-point Likert scale, this study found that disaster preparedness in the community was more motivated by the desire to cope with unexpected challenges (*M* = 4.06, *SD* = 1.24) than the protection of private properties (*M* = 3.51, *SD* = 1.45) (Table 6). In spite of slight variation in participants’ responses, the high mean values recorded from both enquiries denoted a high-risk consideration and perceived preparedness amongst individuals. Although the community’s aggregated mean value (*M*) of risk perception for all hazards was 2.47 (*SD* = 1.27), the aggregated mean value of individual readiness for all hazards was 1.65 (*SD* = 1.00). The low mean value of preparedness recorded by all hazards reflected a considerable difference between perceived probability, personal consequences and perceived preparedness. It also emerged that hazards with a high-risk perception, that is to say earthquake, also attracted more attention and resources.

**TABLE 6:** Descriptive mean summary of readiness for natural hazards.

Natural hazard	Mean	Standard deviation
Earthquake	2.16	1.09
Flooding	2.00	0.93
Tsunami	2.02	1.12
Volcanic eruptions	1.84	0.83
Landslide	1.93	1.01
Coastal erosion	1.80	1.04
Tornado	1.83	0.99

Readiness to cope with natural hazards on 5-point Likert scale:1 = very low, 2 = low, 3 = moderate, 4 = high, 5 = very high.

An enquiry into individual readiness revealed that most people in the community confounded preparedness with response actions. During interviews, it emerged that the community understanding of disaster preparedness was the ability to elevate personal properties and moving to higher ground in case of both flooding and tsunami. Although there was a relationship between risk perception and disaster readiness (Khan et al. 2013), this view did not apply to all hazards under consideration. Whilst there was a relationship between landslide and disaster planning and tsunami and disaster exercise, such a relationship was not found with other hazards under consideration, and none of the hazards influenced personal emergency storage.

The correlation results shown in Table 7 indicate that risk perception and disaster preparedness were not homogenous across hazards. This result supports the conclusion that risk perception does not necessarily influence disaster preparedness (Lindell, Arlikatti & Prater 2009; Paton et al. 2008b). An independent-sample *t*-test did not indicate a significant difference between men and women regarding a household disaster plan, 3-day emergency supply and disaster exercises. The gender of participants did not contribute to general preparedness in the community. Similar results also emerged regarding gender and preparedness for specific natural hazards. These findings aligned with prior studies conducted by Burningham, Fielding and Thrush (2008) regarding awareness and preparedness for a flood in the UK, and by Tekeli-Yesil et al. (2010) in their study about motivation for earthquake preparedness in Istanbul, Turkey. However, Karanci, Aksit and Dirik (2005) found a relationship between gender, disaster awareness and preparedness behaviour regarding earthquake, landslide and floods in Cankiri.

**TABLE 7:** Summary of correlation results of readiness activities and natural hazards.

Natural hazards	Disaster plan	Disaster exercise	Emergency storage
Earthquake	−0.039	−0.012	0.041
Flooding	−0.213	−0.215	−0.067
Tsunami	−0.237*	−0.127	−0.091
Volcanic eruptions	0.076	0.027	0.111
Landslide	0.029	−0.272*	0.074
Coastal erosion	−0.053	−0.076	0.037
Tornado	0.105	0.007	0.154

* , Correlation is significant at 0.05 level (two-tailed).

In understanding the strength and direction of risk and preparedness in the community, this study conducted a Spearman’s coefficient correlation analysis between social variables and indicators of overall preparedness on the one hand (Table 8) and social variables and hazard-specific readiness on the other (Table 9).

**TABLE 8:** Summary of the correlation between social variables and pre-disaster preparedness activities.

Social variables	Disaster plan	Disaster exercise	A 3-day emergency storage
Education	0.089	0.058	0.187
Years in Auckland	0.239*	0.210	0.175
Age	0.292**	0.113	0.311**
Income	0.026	0.010	0.028

Not significant, *p* > 0.05; significant, *p* < 0.05.

* , Correlation is significant at 0.05 level (two-tailed).

** , Correlation is significant at 0.01 level (two-tailed).

**TABLE 9:** Summary of correlation between social variables and hazard readiness.

Dependent variables: Natural hazard	Independent social variables			
	Number of years in Auckland	Education	Age	Income
Earthquake	−0.138	0.061	−0.046	0.223*
Flooding	−0.039	0.145	0.182	0.035
Tsunami	−0.035	0.050	0.134	0.072
Volcanic eruptions	0.020	0.071	0.068	0.150
Landslide	−0.080	0.161	0.228*	−0.044
Coastal erosion	0.030	0.263*	0.253*	−0.001
Tornado	−0.035	0.240*	0.267*	−0.019

Not significant, *p* > 0.05;significant, *p* < 0.05.

* , Correlation is significant at 0.05 level (two-tailed).

A positive relationship existed between the number of years participants had resided in Auckland and the willingness to have a disaster plan for a potential hazard event in Auckland: disaster plan, *r* = 0.239, *p*< 0.05 (two-tailed). Similarly, age of individuals in the community was highly significant with having a disaster plan, *r* = 0.292, *p*< 0.01 (two-tailed); and making provision for a 3-day emergency storage, *r* = 0.311, *p* < 0.01 (two-tailed).

Age was significant in individual readiness for landslide, *r* = 0.228, *p*< 0.05 (two-tailed); coastal erosion, *r*= 0.253, *p*< 0.05 (two-tailed) and Tornado, *r*= 0.267, *p*< 0.05 (two-tailed). The individual level of education was significantly related to individual perceived readiness for coastal erosion and tornado. Previous studies by Finnis et al. (2010) and Roder et al. (2016) have also supported the relationship between education and disaster preparedness. Participants’ income was positively related to the earthquake. The result suggested that household investment in seismic mitigation and retrofitting could improve as income increases (income, *r =*0.223, *p*< 0.05 [two-tailed]). This finding was in line with the prior work of Turner et al. (2003) regarding the risk of an earthquake in California.

## Conclusion

This study has analysed risk awareness and interpretation in the South African community in Auckland. The hazards examined were earthquake, flood, tsunami, volcano, landslide, coastal erosion and tornado. Findings from this study indicate a high level of risk awareness in the community. As hazards, risks of earthquake and flooding that are likely to impact the community and households were consistent. Official information on risk was a critical input into the community’s risk decisions. The influence was because of lack of experience and trust in the official source of information. Whereas trust in official information was influential in risk decisions, role of the media and personality factors of the recipients cannot be overlooked. Although a relationship existed between risk knowledge and preparedness, such a relationship was not linear because of intervening variables between risk and decision. The variables accounted for the low level of disaster preparedness in the community.

Wide disparity in perception amongst hazards was indicative of the level of importance and preparedness of participants attached to respective hazards. In a multi-hazard environment such as Auckland, the importance of preparing for an all-hazard disaster couldn’t be overemphasised because climate change and current complexities are associated with hazards. Whilst the community may not have experienced a disaster, a policy that bridges risk and disaster mitigation would enhance resilience of the community to local hazards. Such policy includes but not limited to community participation in risk identification, defining their vulnerability and formulating strategies for resilience, because communities are more likely to implement mitigation strategies when they participate in the process. The researchers recommend further investigation on how to narrow the gap between risk and resilience behaviour amongst the population that has barely experienced a disaster.
